# Physical and Cognitive Performance of the Least Shrew (*Cryptotis parva*) on a Calcium-Restricted Diet

**DOI:** 10.3390/bs2030172

**Published:** 2012-08-16

**Authors:** Jessica L. Czajka, Timothy S. McCay, Danielle E. Garneau

**Affiliations:** 1 Albany Medical College, 47 New Scotland Avenue, Albany, NY 12208, USA; E-Mail: czajkaj1@mail.amc.edu; 2 Department of Biology, Colgate University, Hamilton, NY 13346, USA; E-Mail: tmccay@colgate.edu; 3 Center for Earth and Environmental Science, State University of New York Plattsburgh, Plattsburgh, NY 12901, USA

**Keywords:** *Cryptotis parva*, calcium, running speed, spatial memory, acid deposition

## Abstract

Geological substrates and air pollution affect the availability of calcium to mammals in many habitats, including the Adirondack Mountain Region (Adirondacks) of the United States. Mammalian insectivores, such as shrews, may be particularly restricted in environments with low calcium. We examined the consequences of calcium restriction on the least shrew (*Cryptotis parva*) in the laboratory. We maintained one group of shrews (5 F, 5 M) on a mealworm diet with a calcium concentration comparable to beetle larvae collected in the Adirondacks (1.1 ± 0.3 mg/g) and another group (5 F, 3 M) on a mealworm diet with a calcium concentration almost 20 times higher (19.5 ± 5.1 mg/g). Animals were given no access to mineral sources of calcium, such as snail shell or bone. We measured running speed and performance in a complex maze over 10 weeks. Shrews on the high-calcium diet made fewer errors in the maze than shrews on the low-calcium diet (F_1,14_ = 12.8, *p* < 0.01). Females made fewer errors than males (F_1,14_ = 10.6, *p* < 0.01). Running speeds did not markedly vary between diet groups or sexes, though there was a trend toward faster running by shrews on the high calcium diet (*p* = 0.087). Shrews in calcium-poor habitats with low availability of mineral sources of calcium may have greater difficulty with cognitive tasks such as navigation and recovery of food hoards.

## 1. Introduction

Chronic acidic deposition, which results from air pollution, increases environmental exposure to toxins and depletes important nutrient cations, including calcium [1,2]. Habitats affected by acidic deposition often have low or reduced abundance of high-calcium invertebrate animals, including snails [3,4,5,6]. Snail shells are an important source of calcium for passerines and reduced snail density may result in increased eggshell deformities and population declines [4,7]. Tree swallows (*Tachycineta bicolor*) experience reduced fitness and altered foraging behavior in areas with low calcium, resulting in longer search times and greater predation risk [8].

Calcium content of invertebrates in forests with calcium-rich soils is greater than invertebrates found associated with calcium-poor soils [9]. Perhaps to compensate for lower calcium availability in invertebrates, passerine birds consume more oak (*Quercus* spp.) buds in areas with low soil calcium. In poorer soils, calcium levels were higher in oak buds as compared to all other invertebrate taxonomic groups, except for spiders [7]. The need to supplement diets with hardwood buds, in areas depleted of calcium, might interestingly exacerbate losses attributed to white-tailed deer (*Odocoileus virginianus*) browsing in northern forests [10,11].

The physiological calcium requirement of birds generally is 10 - 15 times that of mammals [12], underpinning the vast amount of research on avian diet and physiology in habitats with low calcium availability. The use of supplemental calcium by mammals is less well understood. However, a deficiency of dietary calcium may limit reproduction and development among insectivorous bats in nature [13,14,15]. Indeed, periodic deficiencies in dietary calcium generally may exist for mammals that rely on invertebrate foods [14]. Non-volant insectivores, such as shrews, may be more vulnerable to local calcium deficiency than birds and bats of similar size because they are more closely tied to their local habitats. Northern short-tailed shrews (*Blarina brevicauda*) apparently use snails heavily in some regions [16].

Decreased dietary calcium availability has been shown to retard growth [17] and decrease motor performance [18] in laboratory rodents. Female round-eared elephant shrews (*Macroscelides proboscideus*) supplemented with dietary calcium displayed higher density of bone calcium and enhanced reproduction [19]. Limited calcium intake was associated with reduced fecundity in the California vole (*Microtus californicus*) in nature, and females of this species preferentially ate high-calcium foods during the reproductive season [20].

Calcium-deficient diets may impair the cognitive abilities of mammals, potentially reducing their capacity to learn, forage, acquire mates, avoid predators, and navigate efficiently [21,22,23]. Recognition of environmental landmarks, which is dependent upon spatial memory, can have important consequences for survival and reproduction [24]. The retrieval of food hoards depends upon accurate spatial memory. Calcium deficiency has been observed to severely limit the cognition of female Norway (Wistar) rats (*Rattus norvegicus*) [23]; and calcium-dependent protein kinases (PRKCs) are significant predictors of spatial memory and behavior [21,25]. When Norway (Sprague-Dawley) rats were exposed to radiation that impaired bodily PRKC function, memory formation was adversely affected [25].

Our aim was to better understand the implications of calcium depletion on shrews. The least shrew (*Cryptotis parva*) inhabits the forest-floor and consumes invertebrate prey. It is one of the most widespread shrew species in North America [26] and a well-developed laboratory model [27]. More recently shrews have been used as models of bioaccumulation to test environmental changes in terrestrial systems, likely due to their high metabolic rates and constant foraging behavior [28,29]. 

*Cryptotis parva* has a high metabolic rate, making them likely responsive candidates to environmental change [30]. We studied the physical and cognitive performance of least shrews maintained on diets that differed in calcium availability. Because the least shrew is known to hoard food [31], spatial memory might be particularly important in meeting the high energetic requirements of this species in an environmentally sensitive area.

## 2. Materials and Methods

### 2.1. Animal Husbandry and Diet

Our shrews were descendants of a least shrew colony originating from Boone County, Missouri in 1966 [32]. Shrews were marked with passive integrated transponder tags (Biomark, Inc., Boise, Idaho) for unique identification. Least shrews were maintained on a 12:12 L:D cycle and bred throughout the year. Animals were maintained in the Colgate University vivarium on a mixture of laboratory insectivore diet (Lab Diet Advanced Protocol^®^ Insectivore Diet; crude protein ≥ 28.0%, Ca 1.4%), commercial cat food, and spring water. All procedures followed approved Colgate University Institutional Animal Care and Use protocols. 

Twenty shrews were randomly selected from our colony using random number generation and assigned to two dietary calcium groups: a high-calcium group and a low-calcium group. Random selection was continued until there were 5 females and 5 males in each group. Two males from the high-calcium group died early in the experiment due to unknown causes, necropsies were performed and no abnormalities were noted. As a result, data related to these animals were disregarded. All animals were maintained on the same diet, as described above, for two weeks prior to trial implementation and were fed *ad libitum* [31].

Experimental diets were prepared by raising mealworms (Grubco, Inc., Fairfield, Ohio) on chick starter. Mealworms for the low-calcium diet were raised on chick-starter alone; mealworms for the high-calcium diet were raised on chick starter with 8% (by mass) reagent grade CaCO_3_ [33]. Mealworms were raised on these media, along with apple slices for moisture, for >48 h prior to homogenization and storage at −8 °C until use. Calcium concentrations of both diets were analyzed elementally using inductively coupled plasma-atomic emission spectroscopy following wet digestion.

The high-calcium mealworm diet had a calcium concentration that was almost 20 times that of the low-calcium diet (Table 1). The level of calcium in the low-calcium diet (1.10 ± 0.34 mg/g) was comparable to the calcium concentration in a large assortment of adult beetles from Michigan (1.05 ± 0.05 mg/g [34]) and similar to the level of calcium in assorted beetle larvae collected from a site in Herkimer County, New York (3.39 ± 0.87 mg/g, n = 7, unpublished data). The low-calcium diet also was slightly lower than the calcium concentration in our maintenance diet. Shrews were deprived of food for 5 h prior to all trials to increase the motivating effect of a food reward [35]. Mass (g) of shrews at the start and end of the experimental period were recorded.

**Table 1:** Average (± SE) calcium concentration in shrew diets as compared to the base diet. Averages are based on three independent preparations of food made during the experiment.

Calcium (mg/g)	Treatment
4.27 ± 0.68	Base diet
1.10 ± 0.34	Low
19.47 ± 5.05	High

### 2.2. Performance Assays

**Figure 1:**
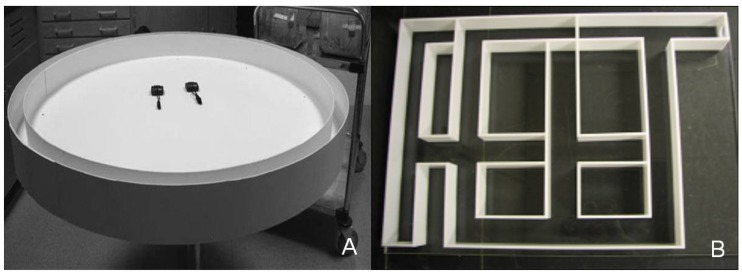
Photographs of the equipment used in the examination of performance of shrews on low-calcium and high-calcium diets: (**A**) Running track; (**B**) Complex maze.

Our running trials and complex-maze assay followed that of Punzo and Chavez [35]. Running speed was measured on a 4 m circular, closed plywood track (Figure 1A). Shrews were placed inside the track using a conical plastic tube for transfer. A 25-mL plastic culture dish, partially filled with mealworms, was placed in front of the plastic tube for reinforcement. When the shrew entered the track, the plastic tube was withdrawn and the animal was coaxed around the track by gentle prodding (no physical contact) with a padded wooden dowel to prohibit exploration [35]. Stopwatches recorded the time necessary to complete one lap of the 4 m track. After completing one lap around the track, shrews were allowed to consume mealworms before being returned to the plastic tube for relocation to the holding cage. The track was disinfected with unscented soap and water between trials to reduce olfactory cues.

Each shrew completed a set of 5 trials, with 5 min of rest between trials, on each of 2 days every 2 weeks. Thus, each shrew completed 10 trials every 2 weeks, for a total of 60 trials over the 10 weeks study (Week 0, Week 2, Week 4, Week 6, Week 8, and Week 10). Shrews were tested in random order, with a new random order determined each testing period. Data were averaged across the 10 trials within a testing period for each animal to provide a single replicate observation for each animal every 2 weeks.

A complex maze (Figure 1B) was constructed following the published diagram in Punzo and Chavez [35], which was used successfully by these authors to assess spatial learning in *C. parva* of different ages. The maze was 45 cm × 60 cm with channels constructed from white acrylic and a clear acrylic top. The maze contained five 5 cm blind alleys and start and goal boxes with removable sliding acrylic gates. The goal box contained a dish filled with mealworms as a reward. Shrews were placed in the starting box for roughly 5 min to allow habituation.

The number of errors was recorded during each trial. An error was recorded when the entire body of the shrew, minus the tail, entered a blind alley [35]. The trial ended when the shrew reached the goal box. Each shrew was subjected to 10 trials every two weeks during the 10 weeks study for a total of 60 trials. Trials were considered subsamples within each 2 weeks period. Shrews were tested in random order as indicated above for running trials. The track was disinfected with unscented soap and water between animal trials to reduce olfactory cues.

### 2.3. Data Analysis

Two-way repeated-measures analysis-of-variance, a test robust to unbalanced design, was used to evaluate the influence of dietary calcium, sex, and diet × sex interaction on each of running speed and maze-error rate. Analyses were performed using SPSS^®^ (version 14.0 for Windows). Residuals were examined for normality after models were fit to the data. The Greenhouse-Geisser correction to degrees of freedom was used for factors in the model involving time [36]. GPower [37] was used to test for effect size on sex and diet treatments.

## 3. Results

### 3.1. Running Track Trial

**Figure 2:**
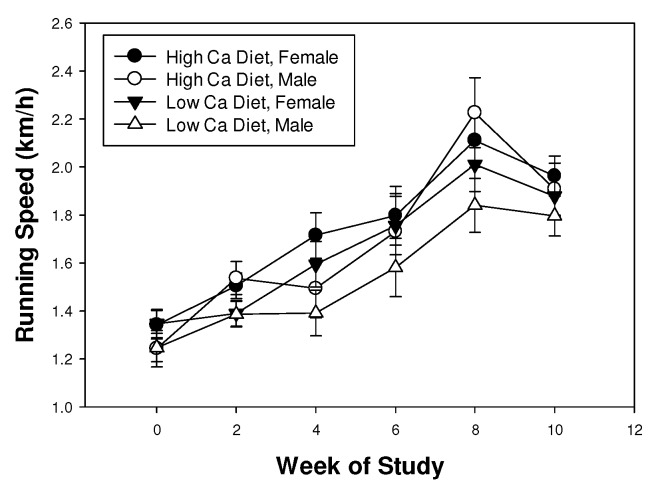
Mean (± SE) running speed of least shrews maintained on mealworm diets with different calcium content.

Shrews ran increasingly faster over the 10 weeks of the experiment (F_3.5,49.1_ = 43.8, *p* < 0.001), presumably as they became more proficient at this assay. Shrews completed the course at approximately 1.2 km h^−1^ at the beginning of the experiment and at approximately 2.0 km h^−1^ at the end (Figure 2). Improvement in performance over time was not affected by diet or sex (all interactions *p* > 0.05). Running speed was not affected by diet (F_1,14_ = 3.4, *p* = 0.087) or sex (F_1,14_ = 2.0, *p* = 0.18), though there was a tendency for shrews to run faster on the high calcium diet (Figure 2). 

### 3.2. Complex Maze Trial

**Figure 3:**
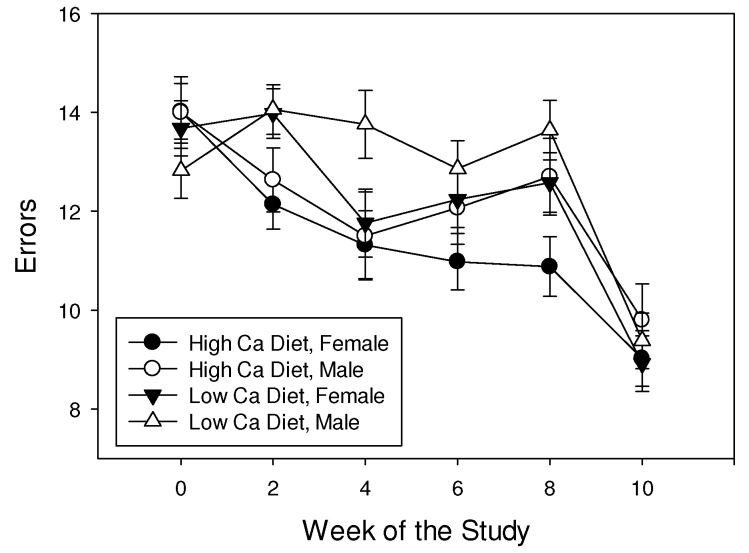
Mean (± SE) number of errors made by least shrews maintained on mealworm diets with different calcium content.

Shrews made fewer errors in the maze trial over time during the 10 weeks of the experiment (F_3.6,49.9_ = 21.7, *p* < 0.001), but the rate of improvement in performance was not affected by diet or sex (all interactions *p* > 0.05). Shrews maintained on a high calcium diet made fewer errors than those maintained on a low calcium diet (F_1,14_ = 12.8, *p* = 0.003; Figure 3). Also, females made fewer errors (11.8 ± 0.13) than males (12.4 ± 0.15; F_1,14_ = 10.2, *p* = 0.006). 

### 3.3. Shrew Mass Fluctuation

Shrews in the low calcium diet lost mass over the course of the experiment with males and females losing 3.46% and 8.56% of their starting mass, respectively (Table 2). Contrastingly, shrews in the high calcium diet gained 2.65% and 0.65% of their body mass, among males and females respectively.

**Table 2:** Average gender-specific mass (g) of shrews in low and high calcium diet treatments.

Treatment	Starting Mass (g) ± SE	Ending Mass (g) ± SE
Females High Ca	6.10 ± 0.30	6.14 ± 0.30
Males High Ca	5.65 ± 0.92	5.80 ± 0.71
Females Low Ca	5.84 ± 0.26	5.34 ± 0.30
Males Low Ca	5.20 ± 0.16	5.02 ± 0.17

## 4. Discussion

To efficiently forage, avoid predators, and reproduce, mammals must properly perceive their environment and recollect the location of foods, safe places, and mates [24]. Healthy diets, complete with normal levels of dietary calcium ensure adequate strength of the musculoskeletal system, as well as proper neurogenesis, particularly in the hippocampus in mammals [38,39]. The hippocampus is an area of the brain associated with learning and sensory reception from the environment [40]. It is in this brain area that the conversion of short-term to long-term memory occurs [40,41]. Various vitamins and minerals are essential to proper hippocampal functioning. In particular, low levels of dietary calcium have been associated with reduction of bone density [42], cardiac disease [43], mood disorders and cognitive deficits [44], in addition to loss of balance [45] in numerous species. This experiment set out to test whether lower levels of dietary calcium affected performance of least shrews in speed and spatial navigation trials. 

Exercise has been shown to negate dietary deficiencies in vital minerals and nutrients [46,47]. Our shrews were run on a track over the course of the 10 week study and increased their speeds, regardless of trial and gender, in all but the last week. Support for this finding comes from rodent treadmill tests where enhanced performance in memory and swimming tasks was observed [46,48]. It is possible that the positive performance effects of regular exercise negated the negative effects of dietary calcium restriction in speed trials. Shrews in the high calcium diet had a tendency to run faster in trials, although not with statistical significance. It is possible that balance, shown to increase with calcium intake and increase locomotor performance, was increased in these animals as they performed this task. Researchers have shown that diets enhanced with whey, calcium, and vitamin D increase both rates of insulin receptor expression in muscles and lipid oxidation [49], as well as reduce inflammatory stress [50], which suggests a fitness benefit to dietary calcium supplementation.

Laboratory maze trials can provide an ecologically relevant way to examine spatial perception and recollection [51]. Least shrews are fossorial animals that inhabit the interface of soil and plant litter in a variety of natural habitats [26]. Researchers have noted that fossorial animals make effective spatial orientation decisions when expending energy constructing tunnel systems and avoiding physiological stressors (e.g., overheating; [52,53]). Shrews likely orient themselves in space using olfactory, tactile, and visual cues [54]. In their natural environment, shrews experience mortality from avian and mammalian predators [55] and are likely most exposed to predation when traveling outside of the nest. Thus, properly recalling the location of food caches, nests, and other resources minimizes travel time and predation risk. Known scatter hoarders such as the Merriam’s kangaroo rat (*Dipodomys merriami*) are more efficient spatial navigators as compared to the Great Basin kangaroo rat (*D. microps*), which shows preference for leaves [56]. Least shrews are known to larder hoard, stowing disabled prey at various distances from their nest depending on quality [31]. Like Punzo and Chavez [35], we found that shrews completed our maze with a decreasing number of errors over time, demonstrating an ability to learn the course of the maze and remember it from one week to the next. Similarly, rats that were fed low calcium diets experienced reduced proficiency in memory and learning tasks, but not motor performance [23], comparable to our findings with least shrews. 

Learning and memory are not synonymous, as learning can occur in numerous ways (e.g., habituation or conditioning) and requires input from sensory modalities. Memory, by contrast, is the storage of information received from the senses [57]. The enhanced performance in speed and maze tasks, noted at the start of our trials, could be the result of a short-term memory response. Two types of memory, specifically declarative and nondeclarative, might also explain the early increase in response of our shrews to trials, as declarative memory results from associations following one trial, whereas nondeclarative memory (learning) results after numerous trial exposures [57]. It is possible that the initial improved response observed in our shrews at the start of trial was the result of declarative memory and the later increase in performance a result of nondeclarative memory. Research on the role of intracellular calcium in learning has been addressed at length [58,59,60,61]; however, few studies have been conducted to support the connection between dietary calcium and memory.

Many female mammals, including shrews, must satisfy large nutritional requirements by foraging away from the nest when offspring are still dependent on lactation [62,63]. Thus, navigational errors might have larger negative consequences for females than for males if these errors delay return to offspring. Females made fewer errors in our maze trials than males; however caution must be taken when interpreting these results due to the small sample size resulting from male-biased mortality during the experiment. We acknowledge that our power to detect treatment effects was hindered by low sample size and high variability among individuals. For example, our statistical power to detect the effect of diet on running speed was estimated at 0.64. Thus, it is likely that work with a greater number of individuals might elucidate additional effects of a low-calcium diet. Most studies have found a male advantage in spatial learning and navigation [64,65,66] and some attribute this to organizational effects resulting from surges in steroid hormones [67,68]. Meta-analyses of gender-specific differences in learning and spatial memory reveal a species-specific difference in performance [69]. Galea *et al*. [70] noted that male deer mice (*Peromyscus maniculatus*) and meadow voles (*Microtus pennsylvanicus*) outperformed females in maze trials. Similarly, reproductive male rats have outperformed reproductive females in both the Morris water maze [71] and in radial arm mazes [72], perhaps because they are generally more active [73].

One suggested explanation for gender difference in performance is that males use not only landmarks, but also geometry as they navigate in land and water mazes, which might give them the advantage in water trials over females [74]. Contrastingly, radial mazes often reward participants in the same location, which would be to the benefit of females who recognize quickly landmark cues. Gender differences in performance in water versus radial arm mazes are known to arise from the reward motivation (*i.e*., food, escape from water), which might be perceived with varied levels of urgency [75]. Other researchers have suggested that outcomes may differ between radial arm and water mazes because the former assesses short-term and long-term reference memory, as opposed to short-term working memory. Radial arm mazes appear to lessen an animal’s stress level by constraining their searches to limit decisions once the first arm selection has been made [76]. Although gender differences are widespread in maze trial performance, we agree with other research that posits the ultimate factor influencing performance is likely stress-induced reduction in neurogenesis, which often negatively affect working memory and recognition of items among group-housed male, not female, rats [77]. Changes in neurochemistry, resulting from increases in estrogen, has been shown to enhance spatial working memory in dry-land radial arm maze trials in females [78]. More research is needed on sex differences in navigation among shrews both in the field and lab.

In the northeastern United States, calcium depletion is occurring in high elevation forests receiving acid rain, and this environmental stressor might reduce viability of populations requiring this nutrient [6,79,80]. In this experiment, the reduction in cognitive performance in least shrews represents a subtle physiological mechanism by which this species might be disadvantaged in calcium-limited environments. Birds have been found to experience reduced rates of reproduction in calcium-limited environments [3,81], and there is growing evidence that acid-induced calcium depletion is associated with the decline of insectivorous migrant songbirds in North America [7,82]. Insectivorous mammals, which also have high calcium requirements during reproduction, could be similarly affected by acid deposition. Our results suggest that in the absence of calcium-rich materials, such as snail shells and bone, shrews might have more difficulty locating food hoards, mates, nests, as well as other ecologically relevant destinations in environments with low calcium availability. 

## 5. Conclusions

When placed on a diet with restricted calcium, which simulated conditions in areas of acid deposition, *Cryptotis parva* were less successful in maze trials than animals maintained on a diet with more calcium. Mammals inhabiting areas with low and declining calcium availability, due to acidic deposition, may experience poor spatial memory and learning. These sublethal effects, which may not be obvious in short-term animal surveys, may nevertheless have negative consequences on reproduction and survival. Our study lends support to the usefulness of shrews as model organisms in behavioral studies. Shrews differ from rodents behaviorally, physiologically, and ecologically. Their high metabolic rate and short generation time may make them particularly useful model vertebrates in studies of environmental change.
